# Directed migration of human neural progenitor cells to interleukin-1β is promoted by chemokines stromal cell-derived factor-1 and monocyte chemotactic factor-1 in mouse brains

**DOI:** 10.1186/2047-9158-1-15

**Published:** 2012-07-31

**Authors:** Yumei Wu, Qiang Chen, Hui Peng, Huanyu Dou, You Zhou, Yunlong Huang, Jialin C Zheng

**Affiliations:** 1 Department of Pharmacology and Experimental Neuroscience, Neuroimmunology and Regenerative Therapy Laboratory, University of Nebraska Medical Center, Omaha, Nebraska, 68198, USA; 2 Department of Pathology and Microbiology, University of Nebraska Medical Center, Omaha, Nebraska, 68198, USA; 3 Department of Pharmacology, School of Pharmacy, The Fourth Military Medical University, Xi’an, Shaanxi, 710032, People’s Republic of China; 4 Center for Biotechnology, University of Nebraska Lincoln, Lincoln, Nebraska, 68588, USA

## Abstract

**Background:**

Neurogenesis, including the proliferation, migration and differentiation of neural progenitor cells (NPCs), is impaired in HIV-1 associated dementia (HAD). We previously demonstrated HIV-1-infected macrophages (HIV-MDM) regulate stromal cell-derived factor 1 (SDF-1) production in astrocytes through Interleukin-1β (IL-1β). Chemokines are known to induce NPC migration; however, it remains unclear how chemokines produced in inflammation regulate NPC migration.

**Methods:**

The secretion of SDF-1 and Monocyte chemotactic preotein-1 (MCP-1) in astrocytes upon IL-1β stimulation was measured by ELISA assay*.* Human NPCs were injected parallel along with IL-1β, SDF-1 or MCP-1 intracranially into basal ganglion 1 mm apart in SCID mice, and immunofluorescent staining was used to study the survival and migration of injected human NPCs.

**Results:**

SDF-1 and MCP-1 are secreted by astrocytes upon IL-1β stimulation in a time-dependent manner. Injected human NPCs survived in SCID mice and migrated towards sites of IL-1β, SDF-1 and MCP-1 injection.

**Conclusions:**

In conclusion, chemokines SDF-1 or MCP-1 secreted by astrocytes in the presence of IL-1β injection are attractive to NPCs injected into SCID mouse brains, suggesting that SDF-1 and MCP-1 play important roles in NPC migration during neuroinflammation.

## Background

HIV-associated dementia (HAD) is a progressive neurological disorder that affects 20 ~ 30% of patients with advanced HIV infection. Neurogenesis, which includes proliferation, migration and differentiation of neural progenitor cells (NPCs), in adults has been observed to be impaired in HAD patients [1]. The histological correlate of HAD is HIV-1 encephalitis (HIVE) which is characterized by reactive astrogliosis, accumulation of activated macrophages, microglial activation, virus-infected multinucleated giant cells, and neuronal damage [2]. Chemokines and chemokine receptors are up-regulated in the brains of patients with HIV and HIVE. Among these, stromal cell-derived factor 1 (SDF-1) [3,4] and monocyte chemotactic protein-1 (MCP-1) [5,6] are documented to be involved in the pathogenesis of HAD. SDF-1, the ligand for CXCR4, has been shown to increase in patients with HIVE [3,4]. Evidence from knockout mouse studies showed that SDF-1 and its receptor CXCR4 are crucial for the nervous system development, especially in directing the migration of NPCs in the developing brain [7-10] and peripheral nervous system [11]. We previously found that SDF-1 increases in HAD patients in an IL-1β-dependent manner [12]. Later studies supported that NPCs migrate to the ischemia region [13] and inflammatory area of the brain [14] in response to SDF-1 secretion.

Cells expressing CXCR4 frequently co-express CCR2 receptor on neurons and astrocytes in the cerebral cortex, hippocampus, and substantia nigra [15,16]. MCP-1, also named CCL2, is the ligand for chemokine receptor CCR2. Evidence has shown that MCP-1 is up-regulated [5] during HIVE and accumulates in the cerebrospinal fluid and brains of immunocompromised patients with HAD and HIVE. The HIV protein Tat elevates the expression of MCP-1 in astrocytes, while MCP-1 enhances virus replication and cell death, and can induce immune cells to migrate into the brain [17,18]. However, the exact role of MCP-1 in the development of HAD and especially how MCP-1 affects neurogenesis remains unclear. MCP-1 also plays a critical role in neuroblast migration after focal cerebral ischemia and induces migration and differentiation of subventricular zone cells after stroke [19], suggesting MCP-1 as a potential mediator for NPC migration under HIV-1 inflammation. Hypoxia-induced astrocytes promote the migration of NPCs via vascular endothelial growth factor, stem cell factor, SDF-1 and MCP-1 upregulation *in vitro*[14], further indicating that both SDF-1 and MCP-1 may play critical roles in NPC migration.

In order to achieve endogenous repair, the first step for NPCs is to migrate to regions of brain injury before differentiating into cells with the correct phenotype and then integrate appropriately into neuronal circuits [20]. However, our understanding as to how all of these processes occur during neurogenesis, and how they can be manipulated for therapeutic advantage is incomplete. NPCs provide precursor cells pools for adult neurogenesis for endogenous repair in the subventricular zone (SVZ) and subgranular zone (SGZ). Cell-replacement of NPCs for repairing brain damage in HAD is a subject of great interest [21,22], holding out the hope of actually reversing neurodegeneration. Given the overview of neurogenesis, migration of NPCs is the critical step. To understand how and which chemokines affect NPC migration in HAD may provide possible ways to reverse HAD. However, the mechanism behind how HIVE inflammation induces NPC migration to repair damaged cells remains unclear. Our hypothesis is that chemokines, SDF-1, and MCP-1, produced by activated astrocytes in response to IL-1β, promote NPC migration.

In this study, we investigated the migration of injected human NPCs in SCID mice brain by injecting human NPCs parallel to chemokines or cytokines. We found that chemokines, SDF-1, and MCP-1, were secreted by astrocytes time- and dose-dependently upon IL-1β stimulation, and human NPCs migrated to SDF-1 and MCP-1 in the SCID mouse model. Our data provide further evidence for the critical roles of SDF-1 and MCP-1 in mediating NPC migration during brain inflammation, thus affecting neurogenesis.

## Methods

### Reagents and materials

Human recombinant SDF-1 and MCP-1 were obtained from R & D Systems (Minneapolis, MN). X-Vivo 15 and neural cell survival factor-1 (NSF-1) were purchased from Lonza (Basel, Switzerland). Fetal bovine serum and N_2_ supplement were from Life Technologies (Carlsbad, CA). N-acetylcysteine, basic fibroblast growth factor (bFGF) and epidermal growth factor (EGF) were obtained from Sigma-Aldrich (St. Louis, MO). Leukemia inhibitory factor (LIF) was purchased from Chemicon (Temecula, CA). Anti-Nestin and anti-GFAPantibodies were purchased from Cell Signaling (Cell Signaling, Danvers, MA). All secondary antibodies and Qtracker565 Cell Labeling Kit were purchased from Life Technologies (Carlsbad, CA).

### Human fetal NPC and astrocyte culture

Human NPCs and astrocytes were isolated from human fetal brain tissue (gestational age 13–16 weeks) from elective aborted specimens in full compliance with University of Nebraska Medical Center and NIH ethical guidelines as previously described [10]. Briefly, NPCs were cultured in substrate-free tissue culture flasks and grown as neurospheres in neurosphere initiation medium (NPIM), which consisted of X-Vivo 15 with N2 supplement, NSF-1, bFGF, (20 ng/ml), EGF (20 ng/ml), LIF (10 ng/ml), and N-acetylcysteine (60 ng/ml). Cells were passaged at two-week intervals as previously described [10]. Human astrocytes were cultured at a density of 2 × 10^7^ cells/150 cm^2^ in DMEM/F12 (Life Technologies, Carlsbad, CA), supplemented with FBS (10%), and an antibiotic mixture containing penicillin, streptomycin, and neomycin (Life Technologies, Calsbad, CA). The adherent astrocytes were passaged by treating with 0.25% trypsin (Life Technologies, Calsbad, CA) after 2 weeks in culture to enhance purity. Astrocyte preparations were assessed by immunocytochemical staining using antibody for GFAP. This process yielded a culture of > 98% pure astrocytes.

### Transwell chemotaxis assay

NPC migration was evaluated using an 8-μm pore size transwell system (Costar, Cambridge, MA) precoated with fibronectin (Sigma-Aldirich, St. Louis, MO) at 5 ng/ml in PBS overnight. Briefly, NPCs were dissociated into single cells and resuspended in X-Vivo 15 at a density of 10^6^ cells/ml. The top chamber of the transwell was loaded with 100 μl of cell suspension and cells were cultured for 12 hours to form an adherent monolayer culture. SDF-1 or MCP-1 was added to the bottom chamber as described concentration in X-Vivo 15. After 12 hours, the membrane of the transwell insert was fixed with 4% PFA in PBS, and cells on top of the membrane were removed with a cotton swab. Cells that migrated to the bottom of the membrane were stained with DAPI (Sigma-Aldrich, St Luis, MO) in PBS at 10 ng/ml. For each insert 10 fields were randomly selected under microscopy at 20 × for imaging, and cell numbers were counted by Image-Pro Plus 7.0 (MediaCybernetics, Bethesda, MD). The cell number of each treated group was normalized to the cell number of the control group to calculate the migration index. Statistical differences were assessed by student *t*-test (*p* < 0.05).

### ELISA assay

To assess the concentrations of secreted SDF-1 and MCP-1 in supernatants of astrocytes upon IL-1β stimulation, we used a sandwich fluorescence ELISA system with modifications to a previously described assay [12,23] for SDF-1 and MCP-1 measurement. In brief, 96-well microtiter plates (Corning, Corning, NY) were coated overnight at room temperature with mouse anti-SDF-1 and anti-MCP-1 monoclonal antibody (4 μg/ml, R & D Systems, Minneapolis, MN) in PBS. Non-specific binding was blocked for 2 h with 1% BSA in PBS. Triplicate samples of cell supernatant (100 μl) or a serial dilution of standards of human recombinant SDF-1 and MCP-1 (R & D Systems, Minneapolis, MN)were applied to the wells and incubated overnight at 4°C. Samples were then incubated for 1 h at room temperature with the biotinylated goat anti-SDF-1 and anti-MCP-1 antibodies (300 ng/ml), followed by 1 h incubation with HRP-conjugated streptavidin (R & D Systems, Minneapolis, MN). After three washes with PBS containing 0.05% Tween 20 (PBST), the final reaction product was detected using QuantaBlu™ Fluorogenic Peroxidase Substrate (Pierce, Rockford, IL). Plates were read by SpectraMax GEMINI (325 nm excitation, 420 nm emission, Molecular Devices, Sunnyvale, California). The sensitivity for this assay was 100 pg/ml for SDF-1. For MCP-1 detection,a chromogen substrate K-bluewas then added at room temperature for color development, and stopped with 1 M H_2_SO_4_. The plate was read at 450 nm to generate standard concentration curves for MCP-1 concentration extrapolation.

### Human NPC and cytokine/chemokines parallel injections in SCID mice

Four-week-old male C.B.-17 SCID mice were purchased from the Charles River Laboratory. All mice were housed in the animal facilities at the University of Nebraska Medical Center. All procedures were conducted according to protocols approved by the Institutional Animal Care and Use Committee (IACUC) of the University of Nebraska Medical Center. Briefly, mice were anesthetized with Ketamine (120 mg/kg) and xylazine (16 mg/kg) by i.p, placed in a stereotaxic apparatus (Stoetling, Wood Dale, IL) for intracranial injection in the right cerebral hemisphere. The animal’s head was secured with ear bars and mouthpiece. An injector with a 10-μl syringe was used for cell injection. NPCs at passage 6–9 were labeled with Qtracker565 Cell Labeling Kit and injected into SCID mice at one site, with IL-1β/SDF-1/MCP-1 (25 ng in 2.5 μl) injected at the other site 1 mm apart from the NPC injection siteinto the basal ganglion of SCID mice, PBS was used as control. Coordinates for inoculation were set as: 0.5-0.8 mm posterior to Bregma, 3.5 mm lateral form the Sagittal midline, a depth and angle of 3.6 mm and 35° from the vertical line. Mice were sacrificed 7 days after injection and brains were immediately removed and then placed in 4% PFA for postfixation. The distance between two injection sites was dived into 5 even sections (2 μm each section). A confocal image was taken for each section.

### Immunocytochemistry

Immunocytochemistry for the detection of NPC was performed as described previously [10]. Briefly, cells were fixed with methanol/acetone (1:1) and then washed with PBS. Cells were incubated with antibody for Nestin (1:100; Sigma-Aldrich, St. Louis, Missouri) overnight and then followed by incubation with goat anti-mouse IgG Alexa Fluor 488 (1:1000). Hoechst 33342 (1:5,000, Sigma-Aldrich, St. Louis, Missouri) was used for nuclear staining. Double immunostaining was examined by an E800 Eclipse microscope (Nikon, Japan). For brain tissue staining, 30 μm-thick sections were permeabilized by 0.3% Triton X-100 (Sigma-Aldrich, St. Louis, Missouri) in PBS for 1 h at room temperature, and then blocked in 10% goat serum for at least 1 h at room temperature. Sections were incubated overnight with anti-GFAP and anti-Nestin antibodies at 4°C overnight with shaking, followed with goat anti-mouse and anti-rabbit IgG Alexa Fluor 488 or 594, and donkey anti-rabbit IgG Alexa Fluor 647 (1:1000) for 1 h at room temperature.

### Confocal examination and image analyses

For fluorescence evaluation of human NPC migration to PBS/IL-1β/SDF-1/MCP-1 injection site, brain tissue was collected on post-treatment day 3 after perfusion fixation with 4% paraformaldehyde in PBS. Immunofluorescent staining was performed on sucrose-processed 30-μm frozen brain sections. Antibody for human specific Nestin (AB5922, Millipore, Billerucam, MA ) was used for detection of human NPC in the mouse brain. Astrocytes detected with antibody for GFAP were also observed to display two injection lines. NPC migration patterns (distance and percentage) were visualized and captured with an Olympus FV500 confocal laser scanning microscope using sequential 488/568/647 nm laser line excitation respectively, and images were imported into Image-ProPlus for quantification. NPC migration distance was determined as the distance between the NPC injection site and the furthest NPC migrating in each slice. At least 500 total Nestin positive cells in each section and five serial slides (30-μm slide) were measured. The migration distance was divided into five even sections and serial confocal images were captured. NPCs in each part were counted and the percentage of the migrating NPCs in each part was calculated as the percentage of the cells in each part to the total number of injected cells (human NPCs).

### Statistical tests

All results were expressed as means ± standard deviation of the mean (SD) or standard error of the mean (SEM). All experiments were done in triplicate or quadruplicate. Data was evaluated statistically by student *t*-test and analysis of variance (ANOVA). Significance was considered to be *p* less than 0.05. To account for any donor specific differences, all experiments were performed with a minimum of three donors.

## Results

### SDF-1 and MCP-1 promote human NPC migration *in vitro*

**Figure 1:**
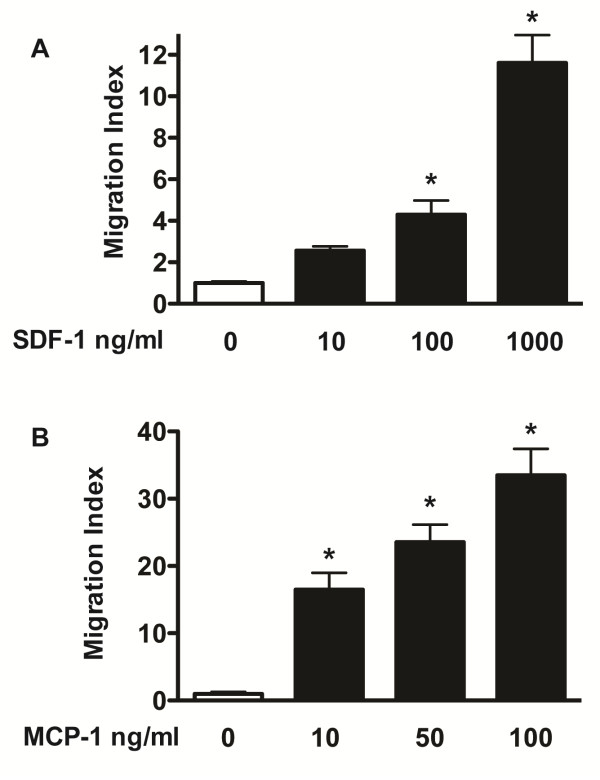
**Human NPC migration to SDF-1 and MCP-1 *in vitro.*** Human NPCs on transwell inserts were incubated under the indicated conditions in the presence of SDF-1 (**A**) or MCP-1 (**B**) at different concentrations as indicated. Migrated cell numbers were counted and normalized to the control group for migration index. * denotes *p* < 0.05.

We first studied the role of the chemokines SDF-1 and MCP-1 in NPC migration using transwell chemotaxis assay *in vitro*. Both SDF-1 and MCP-1 promoted human NPC migration *in vitro* in a dose-dependent manner (Figure 1) as compared to the negative control (basal culture media X-Vivo 15 without chemokine). SDF-1 significantly promoted human NPC migration starting at 100 ng/ml, and MCP-1 significantly promoted human NPC migration starting at 50 ng/ml. With the expression of both chemokine receptors CXCR4 and CCR2 in human NPC [13,24], the *in vitro* data suggested that chemokines SDF-1 and MCP-1 promote NPC migration *in vitro*.

### Human NPC survival and differentiation in SCID mice brain

**Figure 2:**
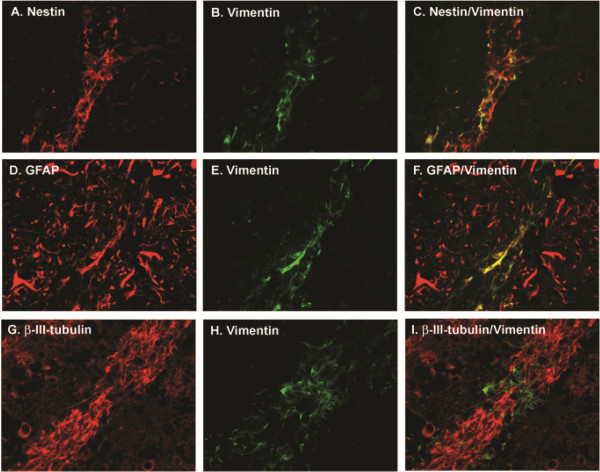
**Human NPC survival and differentiation in SCID mice brain.** Higher magnification (60×) pictures showed injected NPCs expressed Nestin (**A**), weak GFAP (**D**) and Vimentin (**B**, **E** and **H**). Neuronal differentiated cells (β-III-tubulin/vimentinpositive) shown in G did not integrate into the tissue.

NPCs provide a reservoir to replace neurons or glia during conditions of brain injury or disease, and therefore have potential for transplantation therapies in neurodegenerative diseases. To achieve this aim, injected/transplanted NPCs must be able to survive and and differentiate into cells of the correct phenotype. Before determining whether chemokines, SDF-1 and MCP-1, promote NPC migration in SCID mouse brains, we need to study whether injected NPCs can survive and differentiate into proper cell types. We used 5 μm-thick paraffin brain sections to check the survival and differentiation of injected cells by immunostaining with antibodies for NPC marker Nestin and differentiation markers for both neurons and astrocytes, together with human species-specific antibody for Vimentin (a marker for NPC and radial glia) to show the injected human NPC [25]. Figure 2A, B, and C showed that human NPC survived and expressed the NPC markers Nestin and Vimentin, and Figure 2D to I showed NPCs differentiated into astrocytes and neurons, with the expression of NPC specific marker Vimentin to show the human NPC injection site (Figure 2E and H). The integration of NPC and differentiated cells into the host tissue was not observed probably due to the limitation of length of injection time. Higher magnification pictures showed injected NPC expressed Nestin (Figure 2A), weak GFAP (Figure 2D), β-III-tubulin and Vimentin (Figures 2B, E and H). Neuronal differentiated cells (β-III-tubulin) shown in Figure 2G did not integrate into the tissue. These results show that human NPCs survived and differentiated into neurons and astrocytes after injection into the SCID mouse brain.

### The production of SDF-1 and MCP-1 in human astrocytes is stimulated by IL-1β

**Figure 3:**
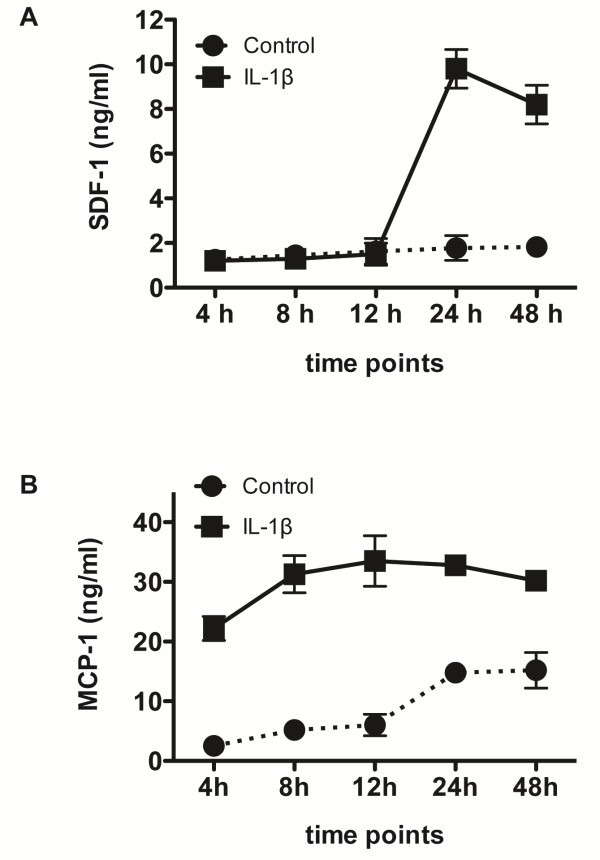
**The production of SDF-1 and MCP-1 in human astrocytes stimulated with IL-1β.** Human astrocytes were stimulated with IL-1β (500 ng/ml), and supernatant was collected at different time points and subjected to ELISA assay. Both of SDF-1 (**A**) and MCP-1 (**B**) showed time-dependent increase upon IL-1β stimulation in astrocytes with a peak time around 24 hour.

Our laboratory previously reported that astrocytes are the major cell type in the brain that is responsible for SDF-1 production and regulation during HIV-1 infection [12]. MCP-1 is another important chemokine that induces immuno-cell migration during inflammation [26]. To investigate the role of SDF-1 and MCP-1 in mediating NPC migration to IL-1β stimulation, we reasoned that both SDF-1 and MCP-1 released by astrocytes upon IL-1β stimulation are the major attractants for NPC migration in diseased brain or during brain inflammation. Since astrocytes are the most abundant cells in the brain and response under cytokine stimulation, it is important to study the release of both chemokines by astrocytes upon IL-1β treatment *in vitro*. Human astrocytes were stimulated with IL-1β (500 ng/ml) at different time points and supernatant was collected and subjected to ELISA assay. Both SDF-1 (Figure 3A) and MCP-1 (Figure 3B) showed time-dependent increase upon IL-1β stimulation in astrocytes with peak time around 24 hour.

### Migration patterns of human NPC after injection along with IL-1β/SDF-1/MCP-1

**Figure 4:**
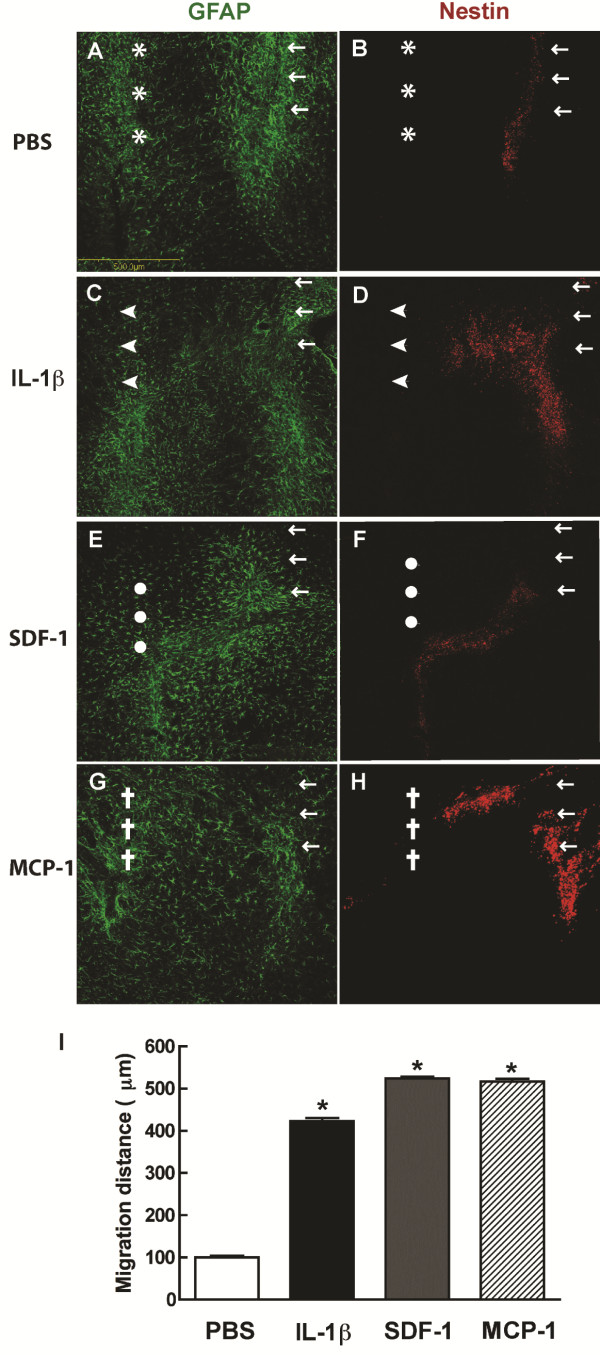
**Migration patterns of human NPC after parallel injections along with IL-1β, SDF-1, or MCP-1.** Human NPCs (**B**, **D**, **F** and **H**) were injected parallel with PBS (**A**), IL-1β (**C**), SDF-1 (**E**), or MCP-1 (**G**), 1 mm apart into the basal ganglia of SCID mice (n = 3 per group). The injection sites were indicated by GFAP (**A**, **C**, **E** and **G**, 10×) and injected human NPCs were visualized by antibody for human Nestin (Figs **B**, **D**, **F** and** H**, 10×). *, ←, ·, and + indicate injection sites. Confocal images illustrated that fewer NPCs migrated towards the PBS injection site (**B**) as compared to NPCs that migrated towards IL-1β (**D**), SDF-1 (**F**), and MCP-1 (**H**) injection sites. The migration distance is showed in **I.**

Injecting/transplanting human cells in a mouse model allows us to track NPC migration, survival, proliferation and differentiation properties *in vivo*. However, antibodies for β-III-tubulin and GFAP are not species-specific. We overcame this obstacle by labeling human NPC with Qtracker565 Cell Labeling Kit to differentiate transplanted human cells from host mouse cells. In the present study, human NPCs were injected into basal gangalia of SCID mice, and 1 mm apart from the NPC injection site, PBS (2.5 μl), IL-1β, SDF-1 or MCP-1 (25 ng in 2.5 μl) were parallelly injected (n = 3 per group). The injection sites were indicated by GFAP (Figure 4A, C, E and G, 10 ×) and injected human NPCs were visualized by human Nestin (Figure 4B, D, F and H, 10 ×). Confocal images illustrated NPC migrated towards the IL-1β (Figure 4D) injection site as well as SDF-1 (Figure 4F) and MCP-1 (Figure 4H) injection sites, whereas fewer NPCs migrated towards the PBS injection site (Figure 4B). The migration distance was determined as the distance between the furthest migrated NPCs and the NPC injection line, as showed in Figure 4I. NPC migration distance was determined with at least 500 total Nestin positive cells in each section of five serial sections (30 μm/section).

### Percentage of human NPC migration induced by IL-1β

**Figure 5:**
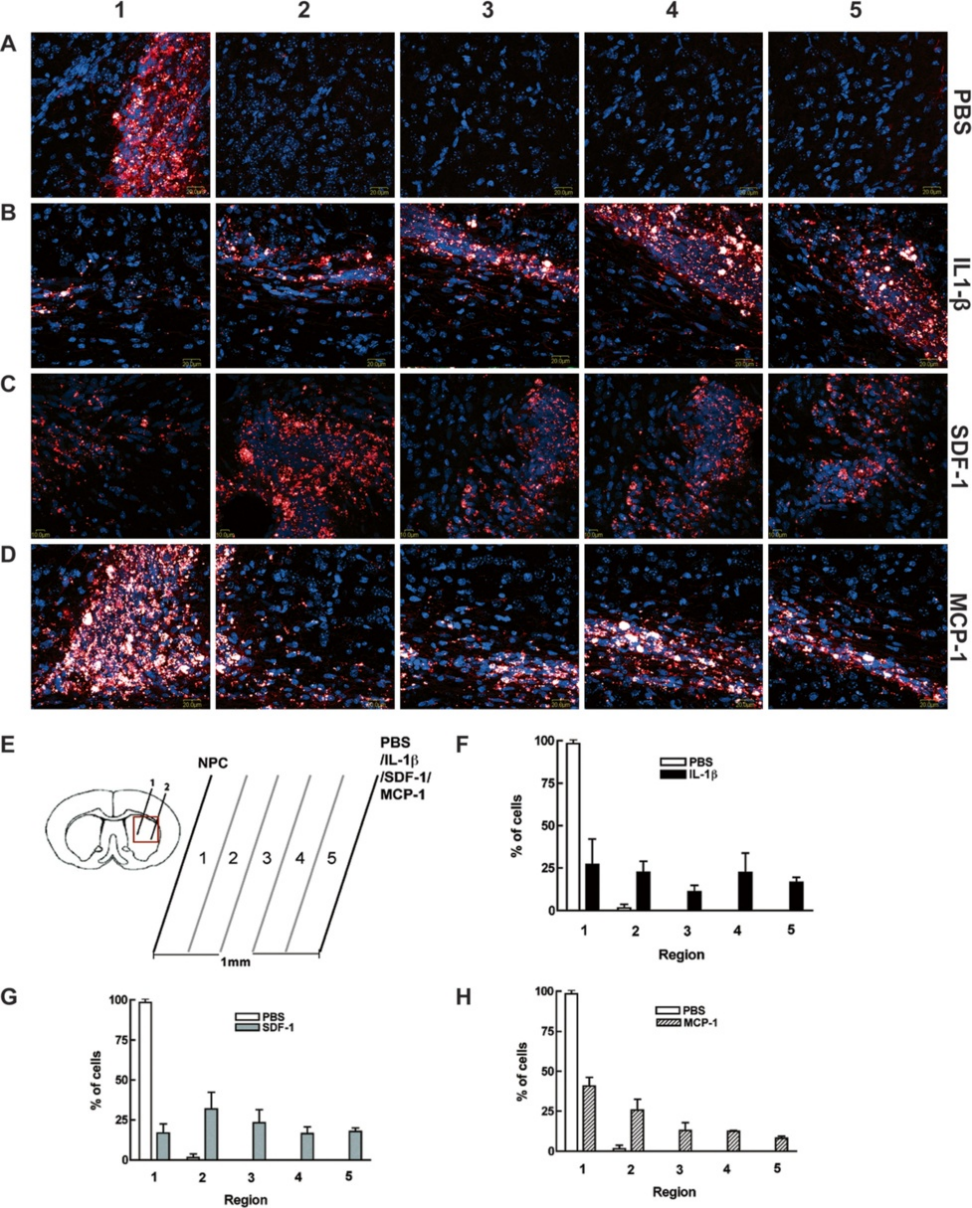
**Percentage of human NPC migration induced by IL-1β.** Human NPCs were injected parallel to PBS, IL-1β, SDF-1 or MCP-1, 1 mm apart into the basal ganglion of SCID mice (n = 3 per group). The distance between the two injection sites was divided into five even sections as diagramed in **E**. **A** slide from each section was picked for serial confocal images for PBS (**A**1-5), IL-1β (**B**1-5), starting from the NPC injection site. Nestin positive cells (red) in each part were counted and the percentage of the migrating NPCs in each part was calculated as the cells in each part/ total migrated cells counted. 83.8% of NPCs in the PBS group were mainly distributed around the injection site (**A**1-2, **F**), and no cells were found beyond the second field (**A**3-5, **F**). NPCs migrated further towards the IL-1β injection site (**B**1-5, **F**). NPCs migrated further to SDF-1 (**C**1-5, and **G**) and MCP-1 (**D**1-5 And **H**) injection sites compared with PBS injection.

IL-1β is an identified factor by which SDF-1 production is modulated *in vivo*. In order to obtain the details about the migration of NPCs induced by IL-1β**,** SDF-1, or MCP-1, NPC and PBS, IL-1β**,** SDF-1 or MCP-1 were injected 1 mm apart into the basal ganglion of SCID mice. The distance between the two injection sites was divided into 5 even sections as diagramed in Figure 5E. Nestin positive cells (red) in each part were counted, and the percentage of the migrating NPCs in each part were calculated as the cells in each part/ total migrated cells counted. 83.8% NPCs in PBS group were mainly distributed around the injection site (Figure 5A1, A2 and F), and no cells were found beyond the second section (Figure 5 A3, A4, A5 and F), suggesting that NPCs mainly stayed in the original injection site, with few cells migrating out of the injection site. The astrogliosis induced by PBS injection was not strong enough to mediate NPC migration. Meanwhile, IL-1β injection led to severe astrogliosis and NPCs migrated further towards IL-1β injection site (Figure 5B1-B5), and 32.8%, 32.4%, 23%, 6.7% and 5.1% cells migrated to IL-1β injection sites in each part (Figure 5F). This suggests IL-1β mediates NPC migration in SCID mice.

### Percentage of human NPC migration induced by SDF-1 and MCP-1

Based on the observation that SDF-1 and MCP-1 produced by astrocytes upon IL-1β stimulation and both chemokines mediated NPC migration, we investigated the difference of the NPC migration induced by SDF-1 and MCP-1. We then injected NPC and SDF-1 or MCP-1, 1 mm apart into the basal ganglion of SCID mice. More NPC migration induced by both chemokines was observed compared with the PBS injection control. The distance between the two injection sites was divided into five even sections as diagramed in Figure 5E. NPCs migrated further towards SDF-1 (Figure 5C1-C5) and MCP-1 (Figure 5D1-D5) injection sites as compared to the PBS injection. 25.4%, 16.2%, 35.6%, 20.2% and 2.5% NPC migrated towards SDF-1 injection sites in each part (Figure 5G), and 40.8%, 25.8%, 12.9%, 12.4% and 8.1% cells migrated towards MCP-1 (Figure 5H) injection site in each part.

## Discussion

In this study, we observed that injected/transplanted NPCs survived and differentiated in SCID mouse brains, and migrated towards the IL-1β injection site and that this may be due to the release of chemokines, SDF-1 and MCP-1 from activated astrocytes which are involved in the pathology upon CNS inflammation induced by IL-1β. To our knowledge, this is the first report to show that the injected human NPCs migrate towards an IL-1β injection site *in vivo*, and that IL-1β may influence neurogenesis during CNS inflammation and disease in brains, and chemokines SDF-1 and MCP-1 released from activated astrocytes are the main factorsponsible for this process.

It is now accepted that neurogenesis takes place throughout life and is capable of replacing neurons, astrocytes, and oligodendrocytes under conditions of brain injury or disease [27-29]. Notably, neurogenesis is indicated to be impaired during brain injury and neurodegenerative disorders by the dysregulation of cytokines, chemokines, neurotransmitters, and reactive oxygen species caused by inflammation and mediated by activated macrophages, microglia and reactive astrocytes [2]. Endogenous neural stem cells produce new neurons to repair in response to injury [22,30,31]; however, neurogenesis has been observed to be diminished in HAD [12,32]. Up to now, our understanding as how all these process occurs, and how neurogenesis can be manipulated towards therapeutic advantages is incomplete.

Chemokines orchestrate the immune system and as well play important roles in activating and recruiting different types of brain cells to a region of injury associated with the pathogenesis of inflammatory neurodegenerative disease [33-36]. The chemokine receptors CCR2, CCR5, CXCR2, CXCR3, CXCR4 and CX3CR1 are constitutively expressed in the human brain, and their expression is enhanced under pathological conditions, including stroke [13,24] and neurodegenerative diseases like HIV-associated dementia (HAD) [4,37] and Alzheimer’s disease (AD) [38]. It is very clear that NPCs express diverse chemokine receptors [39,40] and the NPC migration is promoted by chemokines [41]. To study the role of SDF-1 and MCP-1 in the neurogenesis during CNS inflammation induced by IL-1β, we determined the expression levels of two receptors for both chemokines in NPCs. Our data showed that human NPCs highly expressed CXCR4 and CCR2 by FACS and immuno-staining (data not shown), and this observation kept consistence with previous reports [10,26]. NPC migration towards a single destination is a rapid process and is the essential step not only for the brain development, but also for the pathological mechanisms responsible for CNS disorders [8].

Our laboratory previously demonstrated that SDF-1 secretion from astrocyte stimulated with conditional media from HIV-MDM is through IL-1β, indicating that SDF-1/CXCR4 signaling is important for HIVE pathogenesis [12]. Since CNS inflammation and encephalit is during HIV-1 infection have been shown to decrease neurogenesis, it is possible that deficits of neurogenesis occur in HAD. Taking into consideration that NPCs clearly express diverse chemokine receptors including CXCR4 and CCR2, what are the important roles of chemokines during the progress of HAD? Elucidating the molecular mechanisms that mediate chemokine-induced neurogenesis could facilitate the use of NPCs to replenish neurons for therapeutic transplantation in CNS diseases.

SDF-1/CXCR4 have been shown to be the most important chemokine and chemokine receptor in the CNS development and mediating NPC migration in adults [7-10]. We previously found that SDF-1 increased in HAD patients in an IL-1β-dependent manner [12], so how does this chemokine and its receptor affect NPC migration in the HAD and other brain inflammatory diseases? We have showed that IL-1β, the brain inflammation model we applied, stimulated the production of SDF-1, and both IL-1β and SDF-1 can induce NPC migration. We conclude that SDF-1 is a chemokine that is involved in the impairment of neurogenensis.

We adapted a brain inflammation model with IL-1β injection and determined that IL-1β mediated NPC migration through chemokines SDF-1 and MCP-1. First we observed that injected/transplanted human NPCs survived and differentiated in SCID mouse models with or without cytokine/chemokines injection. This is the basis for a therapeutic purpose of NPC transplantation. We also found that NPCs migrating to IL-1β may through chemokines SDF-1 and MCP-1. It has been demonstrated by our laboratory that SDF-1 secretion from astrocytes mediated by HIV-MDM is through IL-1β, indicating that SDF-1/CXCR4 signaling is important for HIVE pathogenesis [12].

## Conclusions

In this study, we investigated the potential role of IL-1β in inducing NPC migration by stimulating SDF-1 and MCP-1 production by astrocytes, and, together we showed that injected human NPC survived and differentiated into astrocytes and neurons in SCID mice brains. This has shed light on the pathological process of HAD. Our finding of NPC migration to inflammatory cytokine IL-1β also suggests the potential therapeutic application of NPC transplantation in neurodegenerative diseases.

## Competing interests

The authors declare that they have no competing interest.

## Authors’ contributions

Conceived and designed the experiments: YW QC HP YH JCZ. Performed the experiments: YW QC HP HD YZ YH. Analyzed the data: YW YZ YH. Contributed reagents/materials/analysis tools: YZ JZ. Wrote the paper:YW QC HP HD YZ YH JCZ. All authors read and approved the final manuscript.
